# Solving Hard Computational Problems Efficiently: Asymptotic Parametric Complexity 3-Coloring Algorithm

**DOI:** 10.1371/journal.pone.0053437

**Published:** 2013-01-14

**Authors:** José Antonio Martín H.

**Affiliations:** Computer Architecture and Automation, Complutense University of Madrid, Madrid,Spain; Université de Nantes, France

## Abstract

Many practical problems in almost all scientific and technological disciplines have been classified as computationally hard (NP-hard or even NP-complete). In life sciences, combinatorial optimization problems frequently arise in molecular biology, e.g., genome sequencing; global alignment of multiple genomes; identifying siblings or discovery of dysregulated pathways. In almost all of these problems, there is the need for proving a hypothesis about certain property of an object that can be present if and only if it adopts some particular admissible structure (an NP-certificate) or be absent (no admissible structure), however, none of the standard approaches can discard the hypothesis when no solution can be found, since none can provide a proof that there is no admissible structure. This article presents an algorithm that introduces a novel type of solution method to “efficiently” solve the graph 3-coloring problem; an NP-complete problem. The proposed method provides certificates (proofs) in both cases: present or absent, so it is possible to accept or reject the hypothesis on the basis of a rigorous proof. It provides exact solutions and is polynomial-time (i.e., efficient) however parametric. The only requirement is sufficient computational power, which is controlled by the parameter 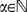. Nevertheless, here it is proved that the probability of requiring a value of 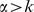 to obtain a solution for a random graph decreases exponentially: 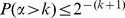, making tractable almost all problem instances. Thorough experimental analyses were performed. The algorithm was tested on random graphs, planar graphs and 4-regular planar graphs. The obtained experimental results are in accordance with the theoretical expected results.

## Introduction

Graph Coloring is one of the oldest and among the most popular Constraint Satisfaction Problems (CSPs) [1]. The study of efficient CSP-solving algorithms is a central topic in Computer Science and Artificial Intelligence because of its wide applicability in many engineering projects, e.g., very-large-scale integration (VLSI) testing, planning and scheduling, timetabling, satellite range scheduling, register allocation, printed circuit testing, and frequency assignment [2]–[4], as well as theoretical physical models, e.g., spin-glasses and the anti-ferromagnetic Potts model [5].

Graph coloring has found applications in life sciences as well, for instance, nucleic acid sequence design has been modeled as a graph coloring problem [6], and in general, combinatorial optimization problems frequently arise in molecular biology: genome sequencing; global alignment of multiple genomes; identification of siblings, cousins, or second cousins through comparison of genomes; finding protein modules containing specified types of proteins; or the computational discovery of dysregulated pathways in human diseases are NP-hard or even NP-complete problems [7], [8]. The computational complexity terminology and concepts (e.g., P, NP, CoNP, NP-completeness, NP-Hardness, certificate/witness, and reductions) can be consulted in the recent books of Arora and Barak [9] and Goldreich [10].

The graph coloring problem involves assigning a number 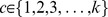 (i.e. a color) to each vertex of a graph such that neighboring vertices are assigned different colors. The problem of deciding whether a given graph can be colored with *k* or less colors is called the *k*-colorability problem.

For *k* = 2, the colorability problem can be solved efficiently. However, for 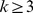, in general, there is no known efficient algorithm to determine whether the graph is *k-colorable* and the problem is NP-complete [11]–[14].

Direct methods for NP-complete problems require the determination of simple properties (e.g. triangle freeness). Such properties impose necessary and sufficient conditions for determining the class of an instance (Yes/No). For the three-color-problem [15], two examples are as follows: phase-transition studies [5], [16]–[19], based on random graphs theory and sharp thresholds [20], [21], and structural-combinatorial approaches based on certain specific parameters such as the existence or absence of particular cycle configurations (see [15], [22]–[25]). One of the foundational results of this approach is the Grotzsch’s 3-color theorem [26]: the triangle-free planar graphs are 3-colorable.

On the other hand, given the intractability of the NP-complete problems, research on approximation algorithms began early (e.g., [2], [27]–[33]). However, even for the approximate case graph coloring remains hard [34]. More strictly, it is NP-hard to even find a 4-coloring of a 3-chromatic graph [35].

A recently developed alternative approach to the classical worst-case computational complexity theory is Parameterized Complexity theory [36], [37]. In Parameterized Complexity, apart from the problem instance itself, there is a parameter (usually an integer) that may be associated arbitrarily with the problem instance, allowing one to study a problem’s complexity with respect to both the size of the input (as in classical computational complexity) and the provided parameter. One of the main virtues of the parametric complexity approach is the concept of fixed-parameter tractability. Here, instead of fixed-parameter complexity, I present an algorithm with *asymptotic* parameter complexity. Two key differences with respect to the past graph coloring approaches are that it is (1) exact and (2) polynomial-time but parametric, while previous algorithms are either exact but low-exponential or polynomial-time but approximate.

A very easy but naive way of coloring a graph is simply to assign sequentially, to each vertex, the first available color. Such an algorithm is known as a greedy coloring algorithm. The base procedure for the proposed algorithm also follows a greedy approach (greedy contraction): sequentially contract two non-neighboring vertices until either a triangle (representing a legal 3-coloring) or a graph containing a 4-clique (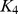) subgraph (a non-3-colorable subgraph) is obtained. However, this greedy algorithm often fails when the graph is 3-colorable since some contractions will unavoidably lead to a 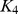 subgraph.

The key idea of the proposed algorithm is a way of determining which contractions will fail, and to avoid them by adding a new edge *G+uv* instead of doing a contraction *G/uv*. Hence, if the graph is 3-colorable, the greedy contraction algorithm will necessarily converge to a triangle, i.e., a legal 3-coloring, and if it is non-3-colorable, a 3-uncolorability certificate will be obtained.

A 3-uncolorability certificate is defined as a sequence of “unavoidable vertex contractions” leading to a graph containing a 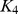. A verifier can efficiently check whether every contraction is “unavoidable”. The possibility of efficiently generating 3-uncolorability certificates is of theoretical interest since 3-colorability is NP-complete, and thus 3-uncolorablity is CoNP-complete. Previous works have studied short proof systems for CoNP-complete problems (e.g., [38], [39]). In particular, for graph coloring, there is a recent work [40] that tries to find graph uncolorability proofs on the basis of consistency checks of CSPs. However, short 3-uncolorability certificates are not possible in general unless NP = CoNP (which remains unknown).

The proposed algorithm has several “good” (desired) key features:

1. The proposed algorithm is exact and runs in polynomial-time; however, it is parametric. Its running time can be controlled (bounded) by means of a simple parameter (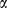, the maximum recursion level), which determines the order of the bounding polynomial. Hence, its complexity is on-demand.
2. If for a given 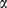 the algorithm is unable to find a certificate then it returns “undetermined”, so that it can be re-run with a higher 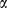 until a solution is obtained, taking into account the computational resources available. Self-tuning the 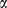 parameter (by using the undetermined return value) gives the algorithm the capability of using only the required computational resources for a particular problem instance, e.g., for almost all planar graphs, it is sufficient to have a value of 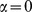 to obtain the right solution, thus obtaining an 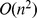 algorithm for the 3-coloring problem in almost all planar graphs.
3. It generates certificates for both Yes and No instances, i.e., either a legal 3-coloring or a 3-uncolorability certificate, and hence, it gives stand-alone indubitable results; hence, it is not necessary to trust neither the correctness of the algorithm itself nor the particular implementation used for recognizing whether the provided solution is correct, since the result can be efficiently verified using only the provided solution (the certificate or witness).

Moreover, from the theoretical point of view, the most important result is the classification of all graphs by the number 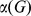: the minimum value of the parameter 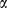 required by the algorithm to obtain a certificate given a particular instance *G* of the 3-coloring problem. A definition assuring that 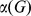 is well-defined is presented in the proof of the main theorem section. This results in important consequences and allows the development of a thorough analysis.

Since for each finite graph *G* there is a corresponding 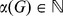 and the algorithm is polynomial-time, and its order depends on 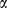,
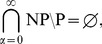
(1)since P also depends on 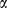.

However, whether for practical or theoretical purposes, the most important thing to be determined is the “speed” of this convergence. Here, as the main theoretical result, it is proved, the non-trivial and highly significant fact, that the probability of requiring a value of 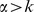 for obtaining a solution decreases exponentially as a function of *k*. This result is formalized as Theorem 1 given below:

### Theorem 1

Let *G* be a random graph; if 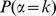 is the probability that 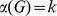 and 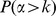 is the probability that 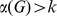 then for all 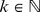:
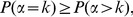
(2)

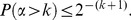
(3)


In the experimental part of this article, the algorithm was thoroughly evaluated using significant samples pertaining to three different graph classes:

1. Random planar graphs [41], [42].
2. Random 4-regular planar graphs [43]–[45].
3. Erdős-Rënyi connected random graphs [20].

An interesting experimental finding is that in all the test cases for planar graphs, it was found that fixing the maximum recursion level to 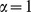 was sufficient to obtain a solution, i.e., exact and efficiently verifiable results were obtained using a polynomial algorithm. This was also the case for 4-regular planar graphs. Furthermore, in the general (random graphs) 3-coloring case experiments, it has been observed that the distribution of 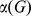 conforms to the theoretical decreasing pattern: the majority of graphs are in 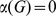 or 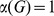, some in 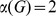, a few in 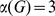, very few in 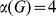, and so on. Indeed, it was not possible to obtain a graph with 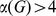 in all the sampled graphs.

### Practical Applicability

The most common methods for dealing with a NP-complete problem when solving a big (intractable by brute force) real problem are: heuristic algorithms, approximate algorithms, randomized algorithms, and fixed-parameter tractability. The presented algorithm introduces a novel type of solution method and presents many new features that are usually absent from the previous approaches.

Suppose that a very critical hypothesis about certain phenomenon needs to be proved, e.g., in a molecular biology study, and that testing such a hypothesis depends on some property of an object, that can be present if and only if the object adopts some particular admissible structure (an NP-certificate) or be absent (no admissible structure for this property), and the problem is not fixed-parameter tractable. Then,

1. None of the standard approaches can discard the hypothesis when no solution is found, since none will give a proof that the problem has no solution, i.e., a proof that there is no admissible structure for this property.
2. Even when a solution exists, heuristic algorithms as well as randomized ones do not guarantee finding a solution, even with extraordinary computational power.
3. Approximate algorithms can give, if lucky, an estimated probability of the existence of this property, including possibly an approximate admissible structure, which is only probably correct.

However, the proposed method solves the problem by providing certificates (proofs) in both cases: present or absent; hence, one can accept or reject the hypothesis on the basis of a rigorous proof, that will be independent of the algorithm itself and the implementation used. Moreover, the proposed method assures an exact solution. The only requirement is sufficient computational power (as in brute force methods). However, here it is proved that, the amount of required computational resources, i.e., the complexity of a problem for the proposed method, is distributed negative exponentially with respect to the problem complexity; hence, the harder a problem is, the lower is its probability of appearance. Thus, the exponential reduction in unsolvable instances makes profitable all investment in computing power.

### Basic Terminology

This article follows the standard graph theory terminology; for general terms and notation, the book of Jensen and Toft [46] and the recent book on chromatic graph theory by Chartrand and Zhang [47] should be consulted. However, some special terms and particular notations are defined below. Unless we state otherwise, all graphs in this work are connected and simple (are finite and have no loops or parallel edges).

The term *random graph* 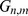 refers to a graph chosen at random (with equal probability) from among all possible graphs with *n* vertices and *m* edges, as defined by Erdős-Rënyi [20].

We refer to *u,v* as a *planar preserving edge* if *uv* is not an edge of *G* and *G+uv* remains planar. A *vertex contraction*, also called vertex identification or vertex merging, is denoted by *G/uv*. Vertex or edge additions and deletions are denoted as follows: *G+u* or *G+uv* and *G-u* or *G-uv*, respectively. A vertex ordering of a graph *G = (V, E)* is a bijection 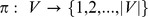, and thus, a set of *n* vertices can be ordered in *n*! different ways.

**Figure 1:**
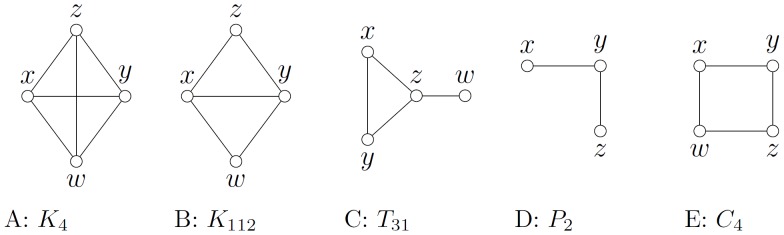
Specially named graphs used in the article. (A) Complete graph 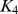. (B) Complete 3-partite graph 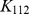. (C) Tadpole graph 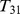, which imposes a binary constraint on 3-coloring: either 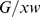 or 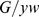. (D) Path of length two 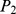. (E) 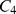 graph that imposes a binary constraint on 3-coloring: either 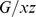 or 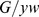.

The specially named graphs used in the article are as follows (c.f. figure 1): complete graph 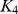, diamond graph, complete 3-partite graph 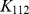 (a 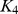 minus one vertex), *tadpole* graph 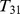, *triangle* graph 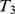, path graph *P* of length two 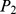, and square graph 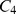.

A *certificate* [9] (or witness [10]) is an efficiently verifiable proof of the correctness of an answer for some given decision problem. For instance, given a graph *G*, a legal 3-coloring of *G* or a short proof that *G* is not 3-colorable are certificates for the 3-colorability problem.

## Materials and Methods

### Definition of the Algorithm

#### Definition 1

Given a graph *G* and a 3-colorable subgraph *H* of *G*, there is an *unavoidable vertex contraction* of *u,v* if the addition of the new edge *uv* to *H* makes *H* not 3-colorable.

Many works has defined the same relation between such a pair of vertices, e.g., “two nodes *u,v* of a given graph *G* are 3-color bound (or simply bound) if *u* and *v* must be assigned the same color in any 3-coloration of *G*” [48]. “Two vertices of a graph are said to be 3-chromatically connected if they are assigned the same color in any 3-coloring of the graph” [15]. Culberson and Gent [17] also use the term “frozen pair”. Moreover, the same relation for *u,v* is called “implicit identity” by the current author, who presents [49] a thorough study of this subject for the general case (k-chromatic and non-planar graphs).

#### Definition 2

Given a non-3-colorable graph *G*, a *3*-*uncolorability certificate W* is a description of a (possibly empty) sequence of unavoidable vertex contractions, *G/uv*, leading to a graph containing 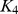, such that either of the following two cases apply:

1. *u,v* are the non-complete vertices of a 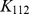 (a diamond) subgraph of *G* or;
2. a nested 3-uncolorability certificate for the graph *G+uv* is provided.

Hence, in order to design an algorithm for obtaining a 3-uncolorability certificate, a method for obtaining such nested certificates should be provided. The proposed algorithm is recursive and uses a parameter 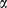 to limit the recursion depth. A very simple sketch of this algorithm is as follows:

**Algorithm: 1** is-3-colorable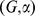:

**1** Contract every *u,v* of a diamond subgraph until no more diamond subgraphs exist; or until the graph becomes the 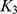 or it contains a 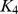 subgraph.

**2** If the graph becomes the 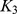 graph then return the current contraction sequence (i.e., a legal 3-coloring).

**3** If 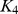 is found then return the current contraction sequence (i.e., a 3-uncolorability certificate).

**4** If the current recursion level 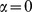 then return “undetermined for the current value of 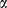.”

**5** For each non-edge *u,v*,

**5.1** If not is-3-colorable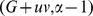 then

**5.1.1** Contract *u,v*.

**5.1.2** Append the nested certificate for *G+uv*.

**5.1.3** Break and continue at step 1.

**6** Return “undetermined for the current value of 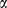.”

**END.**

Now, let us define a greedy 3-coloring algorithm that will serve as the baseline for the derivation of the proposed coloring algorithm.

#### Definition 3

The 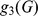 algorithm is a “greedy-contraction” 3-coloring algorithm that sequentially (at each step) selects two non-adjacent vertices *u* and *v* of a graph *G* and contracts them to obtain the graph *G/uv*, while maintaining a list *S* of the vertices contracted thus far.

Hence, if the resulting graph from 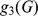 is a triangle (or even a 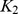) and *S* contains at most three independent sets, these are three (or less) color classes of *G*; hence, *S* is a legal 3-coloring of *G*.

The justification for using such a simple approach, in combination with a more sophisticated way of detecting (and avoiding) the vertex contractions that unavoidably lead to a non-3-colorable graph, is derived from the following lemma (lemma 2):

##### Lemma 2

Given an exact algorithm 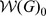 of complexity 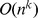 to obtain a 3-uncolorability certificate for any non-3-colorable graph, there is an exact algorithm 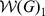 of complexity 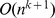 to obtain a 3-coloring of any 3-colorable graph.

##### Proof

Assume 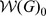 exists. Then, given a 3-colorable graph *G*, apply the greedy 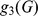 algorithm but avoiding the contraction of every *u,v* such that 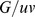 is not 3-colorable, which can be determined in 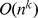 by 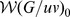. Since *G* is 3-colorable, it will converge (at most) to a triangle graph. Since 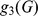 is of complexity 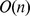 (in every step, at least one vertex will get colored), we obtain a 3-coloring of *G* in 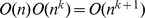.

##### Corollary 3

Hence, if 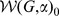 is an exact parametric algorithm, of complexity 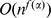, to obtain a 3-uncolorability certificate for any non-3-colorable graph, there is an exact parametric algorithm 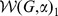 of complexity 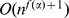 to obtain a 3-coloring of any 3-colorable graph.

Thus, the basic complete coloring algorithm can be described as follows:

**Algorithm: 2** general-3-COL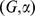:

**1** If not is-3-colorable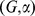 then return 0 and the 3-uncolorability certificate.

**2** While *G* has more than three vertices,

**2.1** Select two non-neighboring vertices *u,v*.

**2.2** If not is-3-colorable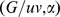 then 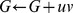.

**2.3** Else, 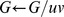.

**2.4** If 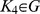, return 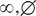.

**3** Return 1 and a legal 3-coloring as the list of contracted vertices.

**END.**

Finally, an automated algorithm can be developed to eliminate the need for specifying the 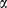 parameter.

**Algorithm: 3** BFS_3COL(*G*):

**1** For 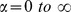,

**1.1** If general-3-COL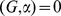, return a 3-uncolorability certificate.

**1.2** If general-3-COL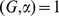, return a legal 3-coloring.

**END.**

### Some Improvements and Special Case Handling

The algorithm is divided into two parts: the decision problem (is-3-colorable) and the coloring algorithms (general-3COL). There are two versions of each of these algorithms: one for planar graphs and the other for non-planar graphs. First, the algorithm for the planar graphs case is described, which is better for understanding the key idea behind the algorithms. Then, this description is generalized for the non-planar graph case.

### Specialization for Planar Graphs

The development of a special algorithm for planar graphs has two main advantages:

1. To take advantage of some special structural constraints of planar graphs (e.g., Grötzsch’s like theorems) that aid the development of more efficient algorithms.
2. To formalize an algorithm for planar graphs that preserves planarity at each step, allowing the development of theoretical studies on the class of planar graphs, e.g., inductive proofs and structure-based proofs.

Now, let us consider the (is-3-colorable) routine. According to Grotzsch’s 3-color theorem [26] (triangle-free planar graphs are 3-colorable), every non-3-colorable planar graph should have {*x,y,z,w*}-tadpole 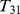 subgraphs (cf. Figure 1B). The key idea is that 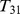 subgraphs impose binary constraints, i.e., either *x,w* or *y,w* must be contracted since 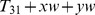 is the 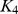 (the same is true for square graphs). Thus, there is no need for Step 5 of Algorithm 1 to check every non-edge but just every 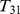 subgraph. Thus, the routine can be performed for each 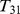 by contracting *G/yw* whenever *G/xw* is not 3-colorable, i.e., when *y, w* is a unavoidable vertex contraction, as shown in the next algorithm:

**Algorithm: 4** is-3-colorable-planar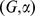:

**1** Contract every *u,v* of a diamond subgraph until no more diamond subgraphs exist; or until the graph becomes the 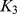 or it contains a 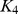 subgraph.

**2** If the graph becomes the 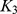 graph then return the current contraction sequence (i.e., a legal 3-coloring).

**3** If 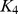 is found then return the current contraction sequence (i.e., a 3-uncolorability certificate).

**4** If the current recursion level 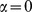 then return “undetermined for the current value of 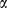.”

**5** For each {*x,y,z,w*}-tadpole 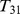 subgraph,

**5.1** If not is-3-colorable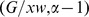 then.

**5.1.1** Contract *yw*.

**5.1.2** Append the nested certificate for *G+yw*.

**5.1.1** Break and continue at Step 1.

**6** Return “undetermined for the current value of 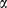.”

**END.**

Now, let us show a planarity preserving coloring algorithm for planar graphs. The idea involves reducing *G* to a planar triangulation by means of the addition/contraction of the planar preserving edges of the planar graph *G*. At the end, if the triangulation has all degrees even, it is 3-colorable [46], [50] and finding a legal 3-coloring is linear-time. Otherwise, the algorithm returns “undetermined”, meaning that 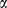 was not sufficient for obtaining a certificate for the input graph. The specialized coloring routine is described next.

**Algorithm: 5** general-3-COL-planar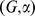:

**1** If not is-3-colorable-planar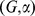 then return 0 and the 3-uncolorability certificate.

**2** While *G* is not a planar triangulation,

**2.1** Select a planar preserving edge *u,v*.

**2.2** If not is-3-colorable-planar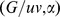 then 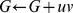.

**2.3** Else, 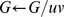.

**2.4** If 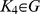, return 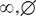.

**3** If triangulation *G* has an odd vertex, return 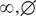.

**4** Return 1 and a legal 3-coloring of *G* in linear time.

**END.**

As can be seen, the graph remains planar at each step, making valid any assumption or structural property of planar graphs in every iteration.

### A Slight Improvement of the Worst and Expected Cases in Non-planar Graphs

For non-planar graphs, a slight modification can be made to improve the worst and the expected case running time of the algorithm. The key idea in this case is to build a complete vertex, i.e., a vertex joined to all the remaining vertices of the graph so that for testing 3-colorability it is sufficient to test 2-colorability of the neighborhood, which can be done in linear time.

**Algorithm: 6** general-3-COL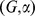:

**1** If not is-3-colorable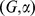 then return 0 and the 3-uncolorability certificate.

**2** Let 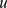 be the vertex with the highest degree of *G*.

**3** While *u* is not a complete vertex,

**3.1** Select a non-neighbor *v* of *u* such that their common neighborhood 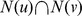 is minimum.

**3.2** If not is-3-colorable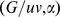 then 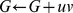.

**3.3** Else, 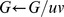.

**3.4** If 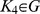, return 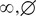.

**3.5** If *N(u)* is not bipartite, return 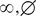.

**4** If *N(u)* is not bipartite, return 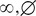.

**5** Return 1 and a legal 3-coloring as the list of contracted vertices.

**END.**

### Proof of the Main Theorem

To formalize the analysis of the algorithm, let us define the following two algorithm specifications:

#### Definition 3

The 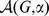 is a parametric-complexity algorithm that computes a function that assigns to a given input graph *G* just one of three possible values: 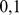, or 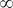, when *G* is, respectively, non-3-colorable, 3-colorable or the algorithm was unable to find a solution for the given value of the 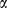 parameter:
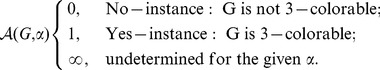
(4)


#### Definition 4

The 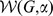 is a parametric-complexity algorithm that computes a function that assigns to a given input graph *G* just one of three possible values: a 3-uncolorability certificate, a legal 3-coloring, or a null value, when 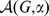 is, respectively, 0, 1, or 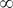.
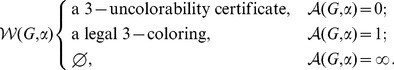
(5)


Since the proposed algorithm is based on a greedy sequential algorithm, it is affected, as well as other greedy sequential coloring algorithms, by the initial vertex ordering; hence, it is not possible to define a function 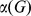 simply as the minimum 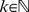 required to obtain a certificate for a particular graph *G* without considering the vertex ordering; for instance, for any 3-colorable graph, it can be shown at least two different vertex orderings 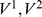 such that for 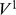, a solution can be found for a value of 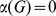, while for 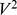, a value of 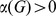 is required. Thus, a solution is to define the function 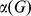 on the basis of the worst-case vertex ordering, and therefore, 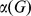 will imply a computational complexity measure.

#### Definition 5

Given a graph *G*, the integer 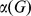 is:

**For** ***G*** **non-3-colorable:** The minimum 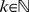 required to obtain a 3-uncolorability certificate, assuming that the ordering of the vertices is the worst case for the is-3-colorable (*G*,*k*) algorithm.

**For** ***G*** **3-colorable:** The value 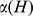 of the non-3-colorable graph 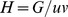 where 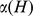 is the maximum among all 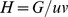 required to obtain a solution for *G*, assuming that the ordering of the vertices is the worst case for the general-3-COL(*G*,*k*) algorithm.
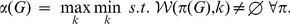
(6)


Now, let us divide the proof of Theorem 1 into two cases:

1. For non-3-colorable graphs, i.e., 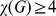.
2. For 3-colorable graphs, i.e., 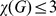.

First, it is proved that for non-3-colorable graphs the cardinality of the set 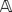 of graphs with 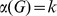 is greater than the set 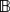 of graphs with 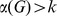.

##### Lemma 4

Let 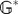 be the set of all graphs and assume (with no loss of generality) that in particular 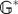 is defined for a maximum number of vertices or edges that exhausts the representation limit of any computational device, i.e., 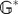 is finite. Let 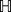, 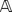, and 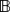 be the sets:
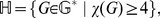
(7)

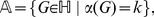
(8)

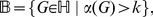
(9)then 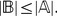
(10)


##### Proof

Since 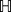 is the set of non-3-colorable graphs, for every graph in 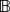, there is at least a distinct graph in 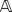 (i.e., an injection): simply take every graph 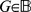 (note that *G* is not in 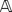 by definition) and join it to the smallest graph 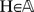. The resulting *GH* graphs are in 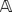, since a 3-uncolorability certificate can be found with 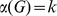, and there is a distinct graph *GH* for each *G*. Moreover, no graph in 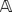 is a subgraph of any graph in 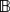. Hence, the cardinality of 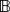 is less than or equal to the cardinality of 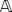.

Therefore, case 1 is proved since 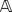 and 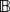 are finite sets and a uniform probability distribution over 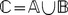 is well defined. Hence, 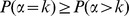 holds for non-3-colorable graphs.

Case 2 can be reduced to case 1 since for 3-colorable graphs 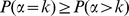 reduces to 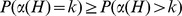 for the worst-case non-3-colorable graph 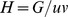 by the definition of 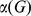 for 3-colorable graphs.

Therefore, on the basis of the lemma 4, Theorem 1 holds for all *k* since 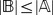 implies that 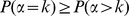, and hence, 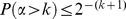. This completes the proof of the main theorem.

### Runtime Analysis of the Algorithm

The average-case complexity, worst-case complexity, and experimental performance of the algorithm are analyzed. The average-case analysis is informally presented as a mean of establishing the theoretically expected behavior over different kinds of instances. The worst-case analysis establishes the order (Big *O*) of the algorithm. Finally, the experimental analysis confronts the algorithm with samples from a series of graph distributions to study its performance and contrast it with the theoretical results.

### Average-case (Expected) Complexity

Table 1 shows the average case (expected) performance of the algorithm with respect to the type of the instance (Yes/No) and the density of the graph, i.e., above/below the phase transition threshold.

**Table 1:** A priori expected performance of the algorithm with respect to 3-colorability and graph density parameters.

KIND	3-COLORABLE	NOT 3-COLORABLE
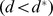 SPARSE	High probability of a short running time due to theexistence of many legal colorings.	High probability of a short running time since almost all non-3-colorable graphs contain a 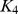 and hence the probability of obtaining a 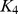 -free non-3-colorable graph decreases rapidly when the average degree falls below the phase transition threshold.
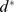	Harder instances.	Harder instances.
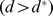 DENSE	High probability of a short running time due to theexistence of many 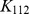 subgraphs that prune thesearch, e.g., graphs tend to be uniquely colorable.	High probability of a short running time due to the existence of many small 3-uncolorability certificates due to the average degree, e.g., too many 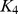 -subgraphs.

Average case (expected) performance of the algorithm with respect to the density of the graph, i.e., above/below the phase transition threshold (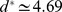) and the type of instance (Yes/No).

In all the cases (except at the phase transition threshold), there is a high probability of a short running time. A priori, it may look that the worst case should occur on the sparse non-3-colorable graphs. This observation is based on the fact that for this class of graphs, it is more complex to obtain a 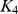 by random edge additions and vertex contractions; nevertheless, some restrictions apply. Since the proportion of non-3-colorable graphs decreases fast below the threshold and almost all non-colorable graphs contain a 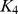, the probability of obtaining a 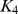-free non-3-colorable graph below the threshold is very small. Moreover, it is known that vertex 3-colorability of a graph with maximum vertex degree three can be determined in polynomial-time [15]. Further, every vertex of maximum degree two can be removed from the graph without affecting the 3-colorability; thus, non-3-colorable sparse graphs are very rare below *c*≲ (this follows from the sharp-thresholds theory).

Hence, in almost all cases, a short running time is expected. By short, I mean significantly shorter than the worst-case upper bound.

### Worst-case Complexity

To determine the computational complexity of the entire algorithm, lets start by analyzing the algorithm from the is-3-colorable routine. This routine admits a special parameter 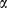 that controls the level of recursive calls. In order to analyze its complexity, the recursion is fixed to 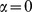, and once the complexity for 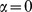 is obtained, the complexity for 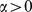 is established.

The is-3-colorable routine depends on the complexity of the contraction step (Step 1). At first sight, the contraction Step 1 has complexity of order 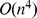 since it explores each 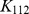 subgraph whose number may increase with an increase in the number of combinations of four elements in the vertices of *G*. However, a relatively in-depth analysis reveals that the algorithm performs a vertex contraction until there is no other 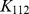. This means that this operation is bounded by the number of edges of the complement of *G*, which has a quadratic 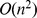 order in the number of vertices. Steps 2 and 3 are absorbed into Step 1. Hence, for 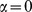,
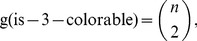
(11)

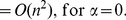
(12)


For 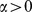, the complexity of is-3-colorable routine also depends on recursive calls inside a for loop through every non-edge that has order 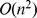; therefore, for 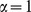, we will have 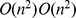.
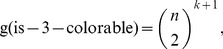
(13)

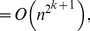
(14)

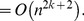
(15)


Thus, on the basis of lemma 2, it can be shown that the complexity of an algorithm that finds a 3-coloring is just one order higher.
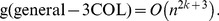
(16)


## Experimental Results

The problem of evaluating algorithms experimentally could be very tricky if tests are performed on “artificial instances”, which may be uncorrelated or isolated from any specific practical application, as claimed by Johnson [51] who proposed a methodological approach to the experimental analysis of algorithms. Nevertheless, there are some lines of research suggesting special distributions of graph instances on which purported NP-complete problem solvers should be evaluated in order to appropriately determine their performances (e.g., [17], [52], [53]).

In the experimental part of this article, the algorithm was thoroughly evaluated over significant samples pertaining to three different graph classes. Each class, and each distribution, has a good justification:

1. Pseudo-random planar graphs [41], [42]. Planarity imposes some interesting structural properties, i.e., the 3-coloring problem on planar graphs is the only unqualified problem that remains open [15] since 1-coloring is trivial, 2-coloring is well characterized, and the maximum chromatic number on the plane is four [54], [55], and at the same time, the determination of 3-colorability of planar graphs is NP-complete [14], [48].
2. Random 4-regular planar graphs [43]–[45]. Even more, the 3-colorability of four-regular planar graphs still remains NP-complete [56], and most importantly, in this class, the average degree is fixed, and hence, the phase-transition phenomenon as defined for random graphs cannot be applied directly in this case.
3. Erdős-Rënyi connected random graphs [20]. Finally, sampling from the Erdős-Rënyi (connected) random graphs distribution gives the necessary theoretical support for evaluating an algorithm in the general case, validating the theoretical bounds and allowing one to obtain results that can be compared against other algorithms in the literature, e.g., the best-performing 3-coloring algorithms proposed in the literature [57].

### Sample Generation Details

Random planar graphs [41], [42] are complex to generate, and their definitions and sampling methods are difficult to implement. Instead, I opted for a relatively simple approach of generating “pseudo random planar graphs”. The procedure involves the generation of a maximal planar graph and the uniform selection of edges from this graph at random (i.e., with equal probability) to create another graph called a pseudo random graph. For this purpose, I used the “Create Random Planar Graph” algorithm implementation used in CATBox [58] for the creation of such random planar graphs. Only one modification was included to avoid generating a considerably large number of graphs containing a 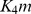 subgraph. The idea involves the generation of a 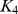-free planar graph during 100 attempts returning the first encountered 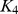-free graph; otherwise, returning the 100th-generated graph.

Random 4-regular planar graphs are also very complex to generate. Here, the procedures described in Refs. [43], [44] and [45] to generate all the 4-regular planar graphs have been used. In particular, Theorem 2 of [45] is used for generating 4-regular planar graphs. However, currently, there is no theory defining a random 4-regular planar graph, so an ad hoc distribution has been specified to balance the proportion of Yes/No instances. The distribution has been obtained by assigning a probability to each graph transformation (see [45]): 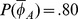, 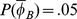, 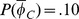, and 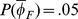.

The Erdős-Rënyi random graph [20], is a very well-known model that allows uniform random sampling of graphs by specifying either the number of vertices and edges, or the number of vertices and a probability for the edges. I followed standard methods to sample from this distribution. The only modification is that the generation of a connected graph is assured by first generating a simple path passing through all the vertices and then adding the remaining edges at random.

The experiments are designed to study the behavior (not just the performance or running time) of the proposed algorithm. For this purpose, there are curves evaluating the algorithm’s performance over a particular graph distribution and also an initial comparative plot against the backtracking algorithm. The use of backtracking is restricted to the study of the algorithm’s scalability since it is not possible to use backtracking consistently beyond the 100 vertex barrier because of its exponential growth. For planar graphs, the planar versions of the algorithm were used, while for random graphs, the improved general versions were used.

All experiments were developed in the Python programming language [59] (version 2.7) using its standard libraries as well as other libraries developed by the current author. Other software used includes the planarity library (http://code.google.com/p/planarity) [60] as well as the above mentioned Graph Animation Toolbox libraries. The experiments were realized on common personal computers (e.g., Core 2 Duo, 2.66 GHz, Windows7 32bits), and no parallelism was used. All time measurements where done in seconds using Python’s time.clock() function (see http://docs.python.org/library/time.html).

### Experiment 1: Scaling Factor Compared Against Backtracking

The first part of the experimental analysis is a comparison between the scaling factors of the proposed algorithm with that of simple backtracking, in order to determine the differences in the behavior of both algorithms. This experiment involved the generation of uniformly random planar graph instances from 10 to 100 vertices (incremented by 1 and generating 100 graphs for each number) and solving each instance with both algorithms. Table 2 shows the parameters of the sample used in the experiment.

**Table 2:** Parameters of the scalability test between backtracking and the proposed algorithm.

Sample property	Value
Sample type:	Random planar graphs.
Sample size:	9000 graphs.
Vertex number:	From 10 to 100 vertices, incremented by 1.
Average degree:	From 2 to 5 edges uniformly distributed.
Group size:	100 graphs per number of vertices.

Random planar graph instances from 10 to 100 vertices incremented by 1 and generating 100 graphs for each number of vertices, i.e., 9000 graphs in total.

**Figure 2:**
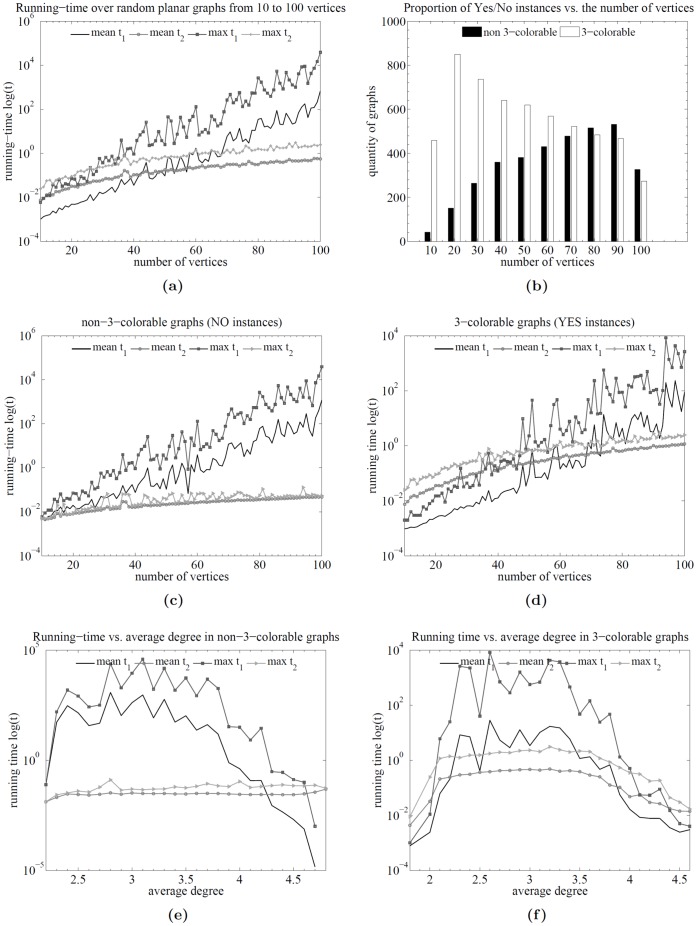
Backtracking vs. proposed algorithm. Runtime analysis of the backtracking (brute force plus heuristic) (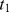) vs. the proposed parametric algorithm (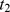) over random planar graphs between 10 and 100 vertices. Plot (a) shows the running times as a function of the number of vertices for both kinds of instance types and for both algorithms. Plot (b) shows the proportion of 3-colorable and non-3-colorable graphs over the total number of graphs per number of vertices. Plots (c) and (d) also show the running times as a function of the number of vertices discriminated by the instance types (yes-or-no) so that subtle differences can be observed. Plots (e) and (f) also show the running times but as a function of the average degree and instance type.

For each algorithm, the mean and maximum (max) running times were recorded as well as some other relevant statistics. Times labeled as 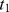 correspond to the backtracking algorithm, while the 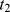 times correspond to the proposed parametric algorithm. Comparative plots are shown in Figure 2; there are six plots from (a) to (f).

Figure 2a shows the running times as a function of the number of vertices for both kinds of instance types and for both algorithms. Figure 2b shows the proportion of 3-colorable and non-3-colorable graphs over the total number of graphs per number of vertices. It can be seen that the distribution tends to be uniform. Figures 2c and 2d also show the running times as a function of the number of vertices but discriminated by the instance types (Yes/No) so that subtle differences can be observed.

The general results indicate that there is a crossing point in which backtracking continues to grow exponentially while the proposed algorithm remains polynomial (cf. Figure 2a at around 50 vertices); hence, a clear difference in the behavior of both algorithms is observed. This difference is clearer in the non-3-colorable instances (cf. Figure 2c) where the maximum running times of the parametric algorithm are relatively low in all the cases. Nevertheless, for the 3-colorable instances (cf. Figure 2d), the difference starts to be clear around graphs on 50 vertices.

Moreover, when running times are compared as a function of the average degree, there is a significant difference in the behavior of both algorithms. For non-3-colorable instances, the parametric algorithm exhibits an almost constant performance (cf. Figure 2e) and a totally uncorrelated curve against backtracking, which on the contrary is very sensitive to the average degree. This difference, although to a slightly minor degree, can also be observed in the 3-colorable instances as shown in Figure 2f.

### Experiment 2: Random Planar Graphs

This experiment involves the generation of uniformly random planar graph instances from 100 to 1000 vertices (incremented by 100 and generating 1000 graphs for each number) and solving each instance with the parametric algorithm. Table 3 shows the parameters of the sample used in the experiment. It is not possible to compare the results against backtracking because of its exponentially increasing running time.

**Table 3:** Parameters of the sample used in the random planar graphs test.

Sample property	Value
Sample type:	Random planar graphs.
Sample size:	10000 graphs.
Vertex number:	From 100 to 1000 vertices, incremented by 100.
Average degree:	From 2 to 5 edges uniformly distributed.
Group size:	100 graphs per number of vertices.

Uniformly random planar graph instances from 100 to 1000 vertices incremented by 100 and generating 1000 graphs for each number of vertices, i.e., 10000 graphs in total.

**Figure 3:**
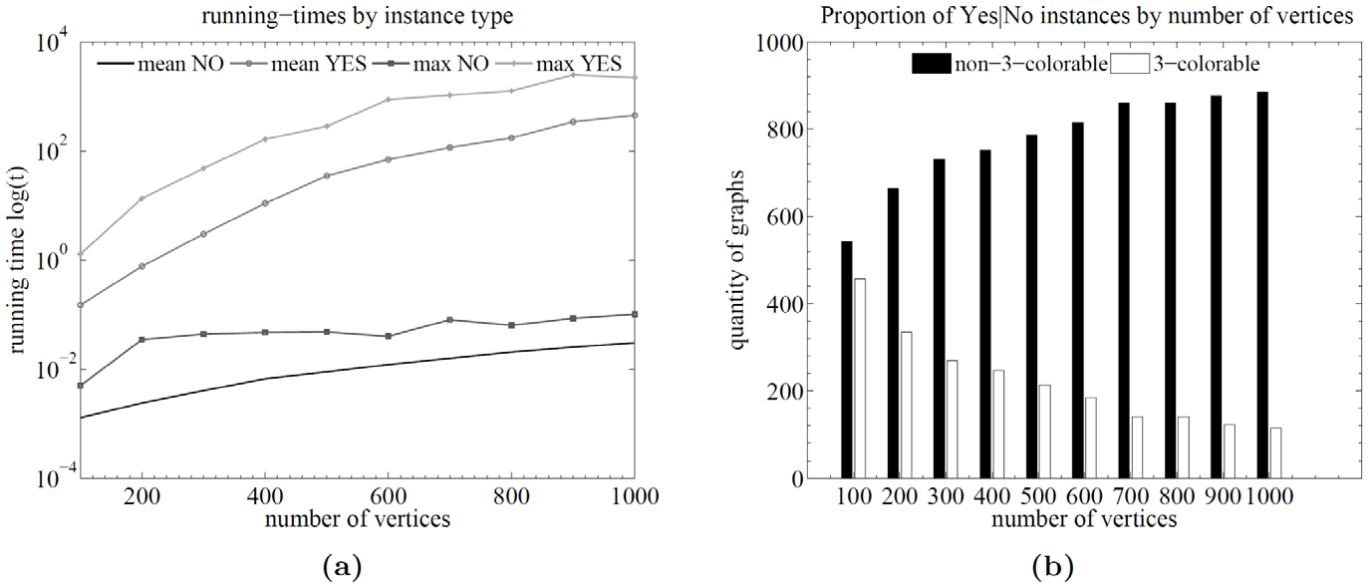
Results for planar graphs. Runtime analysis over random planar graphs between 100 and 1000 vertices (incremented by 100 and generating 1000 graphs for each number). Plot (a) shows the running times as a function of the number of vertices for both kinds of instance types. Plot (b) shows the proportion of 3-colorable and non-3-colorable graphs over the total number of graphs per number of vertices.

**Figure 4:**
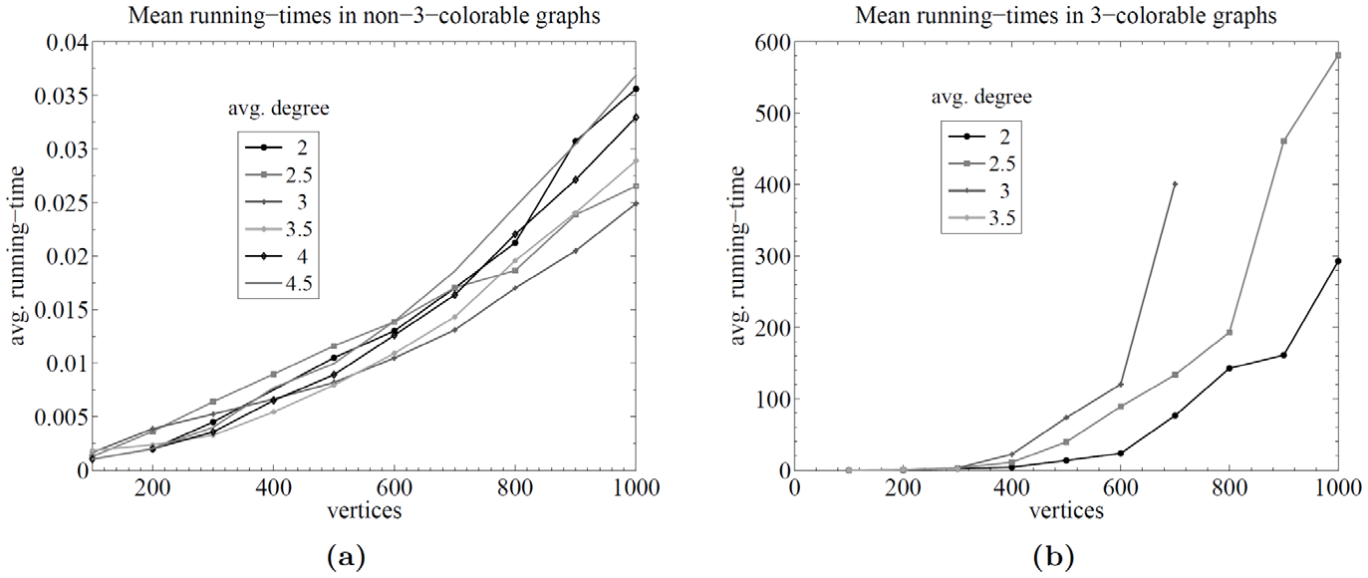
Results for planar graphs. Runtime analysis over random planar graphs considering instance type and average degree. Plots (a) and (b) also show the running times as a function of the number of vertices but discriminated by the average degree.

For each instance type (Yes/No), the mean and maximum (max) running times where recorded, as well as some other relevant statistics. Comparative plots are shown in Figures 3 and 4.

Figure 3a shows the running times as a function of the number of vertices for both kinds of instance types. Figure 3b shows the proportion of 3-colorable and non-3-colorable graphs over the total number of graphs per number of vertices. It can be seen that the distribution is far from uniform. Figures 4a and 4b also show the running times as a function of the number of vertices but discriminated by the average degree.

The results indicate that there is a difference in running times depending on the instance type (cf. Figure 3a). This difference is expected since the proposed algorithm returns earlier (without entering the main loop) when a 3-uncolorability certificate is found. Even in the case when the average degree is considered, the difference is high (cf. Figures 4a and 4b).

### Experiment 3: Random Planar 4-regular Graphs

In this experiment, graphs were sampled from an ad-hoc distribution over the 4-regular planar graphs. The samples were used for generating graph instances from 100 to 1000 vertices (incremented by 100 and generating 1000 graphs for each number) and solving each instance with the parametric algorithm. Table 4 shows the parameters of the samples used in the experiment.

**Table 4:** Parameters of the sample used in the random planar 4-regular graphs test.

Sample property	Value
Sample type:	Random planar 4-regular graphs.
Sample distribution:	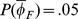 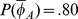 , 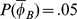 , 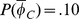 and
Sample size:	10000 graphs.
Vertex number:	From 100 to 1000 vertices, incremented by 100.
Degree:	Fixed: 4-regular graphs.
Group size:	1000 graphs per number of vertices.

Graphs are sampled from an ad-hoc distribution over the 4-regular planar graphs. The sample consist of graph instances from 100 to 1000 vertices incremented by 100 and generating 1000 graphs for each number of vertices, i.e., 10000 graphs in total. For the exact meaning of each graph transformation operation, i.e., 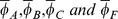 see Ref. [45].

**Figure 5:**
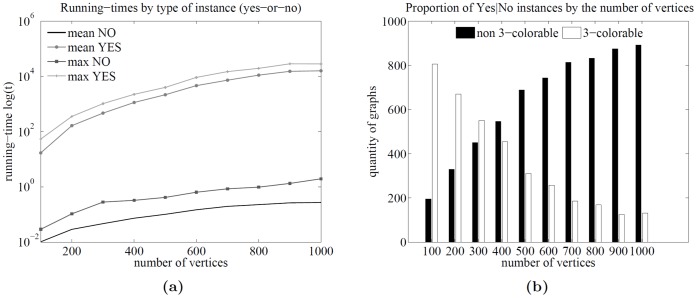
Results for 4-regular planar graphs. Runtime analysis over random 4-regular planar graphs between 100 and 1000 vertices. Plot (a) shows the running times as a function of the number of vertices for both kinds of instance types. Plot (b) shows the proportion of 3-colorable and non-3-colorable graphs over the total number of graphs per number of vertices.

For each instance type (Yes/No), the mean and maximum (max) running times were recorded, as well as some other relevant statistics. Comparative plots are shown in Figure 5. Figure 5a shows the running times as a function of the number of vertices for both kinds of instance types. Figure 5b shows the proportion of 3-colorable and non-3-colorable graphs over the total number of graphs per number of vertices; it can be seen that the distribution is far from uniform.

The results indicate that there is a very significant difference in running times depending on the instance type (cf. Figure 5a). This difference was expected since the proposed algorithm returns earlier when a 3-uncolorability certificate is found. Again, the observed difference is high.

### Experiment 4: Erdős-Rënyi Random Graphs

**Figure 6:**
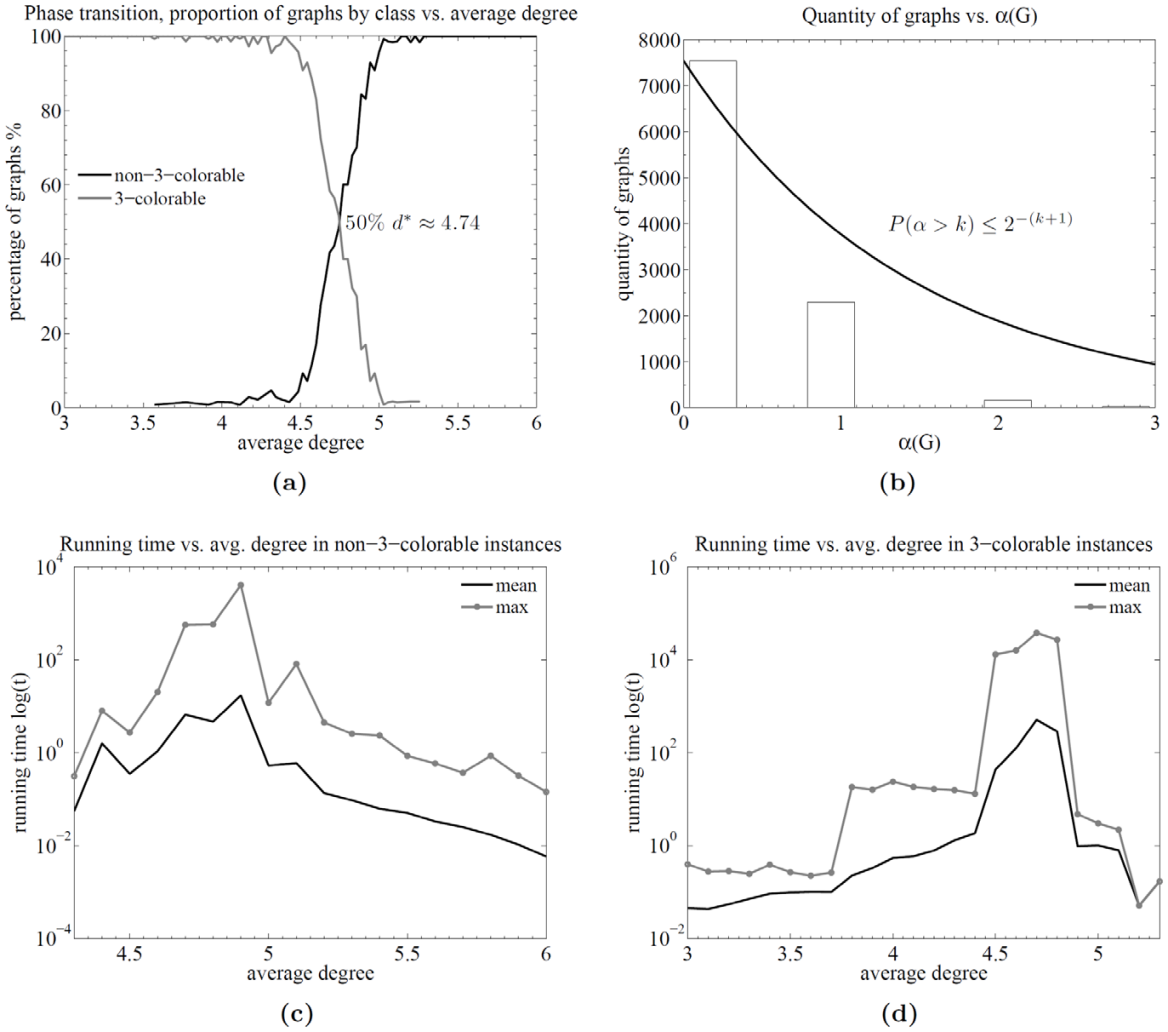
Runtime analysis of the algorithm for random graphs. The behavior of the proposed algorithm over the well-known Erdős-Rënyi random graphs distribution. Plot (a) shows the quantity of 3-colorable and non-3-colorable graphs as a function of the average degree, i.e., a phase transition plot, in this case, occurring at around 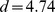. Plot (b) shows the quantity of graphs corresponding to each 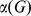 value. As predicted by the theory, the proportion of graphs decreases exponentially as a function of 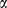 below the line of 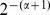. Plots (c) and (d) show the running times as a function of the average degree.

In the last experiment, graphs were sampled from the well-known Erdős-Rënyi random graphs distribution. The samples were graph instances of 100 vertices, generating in total 10000 graphs, and solving each instance with the parametric algorithm. Table 5 shows the parameters of the sample used in the experiment. For each instance type (Yes/No), the mean and maximum (max) running times were recorded, as well as some other relevant statistics. Comparative plots are shown in Figure 6.

**Table 5:** Parameters of the sample used in the random graphs test.

Sample property	Value
Sample type:	Erdős-Rënyi random graphs.
Sample size:	10000 graphs.
Vertex number:	Fixed: 100 vertices.
Average degree:	From 3 to 6 edges uniformly distributed.

Graphs are sampled from the well-known Erdős-Rënyi random graphs distribution. The sample consist of graph instances for 100 vertices, generating in total 10000 graphs. For each number of vertices, the average degree is varied from 3 to 6.

Figure 6a shows the quantity of 3-colorable and non-3-colorable graphs as a function of the average degree, i.e., a phase transition plot, in this case, occurring at around *d* = 4.74. It should be noted that this phase transition is for the *connected* random graphs and not for standard random graphs, which can contain many components, thus affecting the phase transition threshold. Figure 6b shows the quantity of graphs corresponding to each 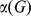 value. As predicted by the theory, almost all graphs have 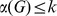 for some integer *k*, and the proportion of graphs decreases exponentially as a function of 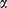 clearly below the line of 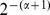. These results confirm the established theoretical bounds.

Figures 6c and 6d show the running time as a function of the average degree. It can be observed that in the random graphs case, the difference in running time is not as high as the difference observed in the planar graphs case. Further, there is a difference in the location of the harder instances for each kind of instance type: the harder instances for the non-3-colorable case are located around an average degree of 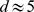 while, in the 3-coloring case, they are located slightly below an average degree of 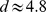. Although the numbers seems to be very close, the shapes of the running-time curves are not. The shape of the running-time curve in Figure 6d falls sharply after 5, while the shape of the running-time curve in 6c does not. This may indicate that there is a true difference in the location of the harder instances depending on the type (Yes/No) of the instance, and (to the best of my knowledge), there are no other works identifying a separation of a complexity threshold on the basis of the type of instance.

As observed in the experiments, the value of 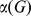 was directly correlated with the average degree *d* = 2*m/n* (*m* = edges, *n* = vertices); hence, near the phase transition threshold (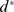) [18], [19], the probability of a relatively high value of 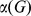 increases.
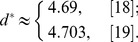
(17)


However, many interesting questions remain open; e.g.,

- What is the exact distribution of *α*(*G*) in random graphs?
- Apart from the average degree, what other parameters are related to *α*(*G*)?
- Given an arbitrary input graph *G*, can the *α*(*G*) value be predicted (exactly or approximately) and what is the best possible approximation to *α*(*G*)?
- As for the chromatic number χ(*G*), is there any (efficient) graph construction mechanism that allows the generation of graphs with arbitrarily large *α*(*G*)?

## Discussion

In this article, an asymptotic parametric exact 3-coloring algorithm has been presented. This is (to the best of my knowledge) the first algorithm of its kind for the 3-coloring problem.

The maximal complexity of the algorithm is controlled by the parameter (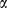) that bounds the recursion depth and determines its running time. The algorithm relies on the efficient search of 3-uncolorability certificates. Here, a formal definition of the 3-uncolorability certificate has been introduced. This is the central theoretical concept that allowed the development of the proposed algorithm. The definition of the 3-uncolorability certificate presented here is (to the best of my knowledge) the first one that is formally presented and the most naturally related to the 3-coloring problem.

A very significant feature of 3-uncolorability certificates is that it is possible to obtain them from small subgraphs of a particular graph, indeed, as small as four vertices (i.e. by finding a 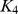 subgraph). Hence, an interesting theoretical analysis that should follow is to study the behavior of 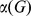 on 4-critical graphs since in this class, there is no subgraph with chromatic number four, and hence, finding unavoidable vertex contractions may be hard (e.g., see Ref. [53] for a good initial development of this idea). Hence, a classification of 4-critical graphs on the basis of 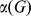 can lead to very significant results.

There is an interesting symmetry between coloring and uncolorability certificates:

- In order to show that a graph is 3-colorable, it is sufficient to encounter just one legal coloring; nevertheless, any legal coloring must assign a color to all the vertices of the graph without violating any constraint since it remains hard to determine if a partial coloring is extensible to all the vertices of the graph.
- Instead, in order to show that a graph is not 3-colorable, one needs to verify that none of the possible 3-colorings is a legal one; nevertheless, for obtaining a 3-uncolorability certificate, it is sufficient to encounter just one non-3-colorable subgraph (e.g., a 4-critical subgraph), i.e., a small graph.

Thus, while for considerably large graphs, just verifying a legal 3-coloring can be complex in practice, it remains practical (at least in theory) to determine 3-uncolorability even for such graphs.

Hence, in principle, finding uncolorability certificates can be assumed to be at least of the same kind as finding colorings. Thus, there should not be any problem in the development of 3-coloring algorithms on the basis of a search for 3-uncolorability certificates that eventually reach the same level of sophistication and performance as its coloring-based peers.

Moreover, if the algorithm is used as a heuristic, e.g., to test whether a solution can be found quickly (“just by chance”) with a relatively low (efficient) 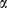, the algorithm will search for both 3-colorings and 3-uncolorability certificates at the same time, in clear contrast with the use of backtracking, greedy-based, and randomized 3-coloring algorithms. Further, this feature is particularly important as its consideration ensure that it is not necessary to trust the correctness of the algorithm itself or the particular implementation used in order to recognize that the provided solution is correct since the result can be efficiently verified using just the solution provided, i.e., a legal 3-coloring or a 3-uncolorability certificate.

The developed theoretical analysis guarantees some good features of the proposed algorithm. The most important one, for both practical and theoretical purposes, is that while the algorithm relies on the value of 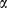 to be able to find a certificate, the probability that 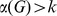 decreases at the rate 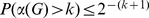, e.g., for *k* = 19, there is less chance than one in a million of not obtaining a solution with the proposed polynomial algorithm (i.e., probability of success = 0.999999), assuming that the input is a random graph.

Thus, while certainly beyond some value of 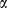, the running times would become prohibitive given the current state of the computing machinery, the developed algorithm scales polynomial, and the probability of obtaining a solution (success) grows exponentially with an increment of 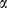. Hence, any step (i.e., any investment) in computing power technology will lead to a huge (exponential) growth of the class of tractable 3-coloring instances, as well as CSPs in general.

Perhaps, it could be the case that we can achieve at least a “technological tractability”? I.e., a guaranteed number of instances such that almost all computational problems of practical interest could be solved for 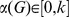 for some integer *k*?

It should also be observed that increasing 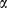 as the result of technological progress implies that 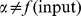, i.e., 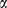 is not a function of the input. Does technological progress imply a polynomial algorithm for 3-colorability?

In addition, since 3-colorability is NP-complete and to each graph corresponds a unique 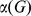, a classification based on 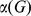 of all the NP-complete problem instances can be done by a reduction of each problem instance to a 3-coloring instance *G* such that 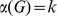 for some 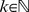.

However, can we define NP as follows? Let us define 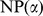 as the class of problems in NP that are also in P for some particular value of 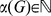. Then,
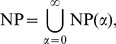
(18)i.e., can then NP be defined as the infinite union of problems in P?

Finally, even determining the infiniteness of 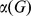, is there, as in the case of the maximal degree four, 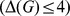; a 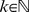 such that determining 3-colorability over a class of graphs with 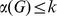 is still NP-complete, i.e., P = NP?

In the maximal-degree case, we know that 3-colorability restricted to 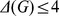 is still NP-complete. Nevertheless, the problem is to determine whether a polynomial algorithm exists or not.

On the contrary, in the finite-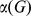 case, we know that 3-colorability restricted to 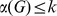 is in P. Nevertheless, the problem is to determine whether it is NP-complete for a class of graphs and finite 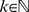.

### Reproducibility Note

The working source-code of the algorithm and all the software libraries needed to appropriately use and experiment with the algorithm have been released and are available at the publisher’s website.

Furthermore, there is a web application that implements the algorithm inside the Google App Engine cloud computing framework. The users can visit the site and test the algorithm at the following web address:

- http://graph-coloring.appspot.com

The web coloring application just asks for a file where a graph is defined following the plain text version of the simple edge-list according to the DIMACS standard format specification, such as the.col files in the Graph Coloring Instances web:

- http://mat.gsia.cmu.edu/COLOR/general/ccformat.ps
- http://mat.gsia.cmu.edu/COLOR/instances.html
